# Apoptosis-Inducing Factor Regulates Skeletal Muscle Progenitor Cell Number and Muscle Phenotype

**DOI:** 10.1371/journal.pone.0027283

**Published:** 2011-11-04

**Authors:** Anne-Sophie Armand, Iman Laziz, Dounia Djeghloul, Sylvie Lécolle, Anne T. Bertrand, Olivier Biondi, Leon J. De Windt, Christophe Chanoine

**Affiliations:** 1 Centre d’Etude de la Sensori-Motricité, UMR 8194 CNRS, Université Paris Descartes, Centre Universitaire des Saints-Pères, Paris, France; 2 The Hubrecht Institute and Interuniversity Cardiology Institute Netherlands, Royal Netherlands Academy of Sciences, Utrecht, The Netherlands; 3 Department of Cardiology, Cardiovascular Research Institute Maastricht, Maastricht University, Maastricht, The Netherlands; University of Frankfurt - University Hospital Frankfurt, Germany

## Abstract

Apoptosis Inducing Factor (AIF) is a highly conserved, ubiquitous flavoprotein localized in the mitochondrial intermembrane space. In vivo, AIF provides protection against neuronal and cardiomyocyte apoptosis induced by oxidative stress. Conversely in vitro, AIF has been demonstrated to have a pro-apoptotic role upon induction of the mitochondrial death pathway, once AIF translocates to the nucleus where it facilitates chromatin condensation and large scale DNA fragmentation. Given that the *aif* hypomorphic harlequin (*Hq*) mutant mouse model displays severe sarcopenia, we examined skeletal muscle from the *aif* hypomorphic mice in more detail. Adult AIF-deficient skeletal myofibers display oxidative stress and a severe form of atrophy, associated with a loss of myonuclei and a fast to slow fiber type switch, both in “slow” muscles such as soleus, as well as in “fast” muscles such as extensor digitorum longus, most likely resulting from an increase of MEF2 activity. This fiber type switch was conserved in regenerated soleus and EDL muscles of *Hq* mice subjected to cardiotoxin injection. In addition, muscle regeneration in soleus and EDL muscles of *Hq* mice was severely delayed. Freshly cultured myofibers, soleus and EDL muscle sections from *Hq* mice displayed a decreased satellite cell pool, which could be rescued by pretreating *aif* hypomorphic mice with the manganese-salen free radical scavenger EUK-8. Satellite cell activation seems to be abnormally long in *Hq* primary culture compared to controls. However, AIF deficiency did not affect myoblast cell proliferation and differentiation. Thus, AIF protects skeletal muscles against oxidative stress-induced damage probably by protecting satellite cells against oxidative stress and maintaining skeletal muscle stem cell number and activation.

## Introduction

Mitochondria are the main source of cellular energy production and defects in mitochondrial function is linked to a variety of inherited human disorders, including cardiomyopathies and myopathies. Additionally, age-related, acquired diseases including neurodegenerative disorders such as Alzheimer’s disease, Parkinson’s disease, amyotropic lateral sclerosis (ALS), cardiovascular disease and skeletal muscle wasting, may be associated to excessive oxidative stress, which can result from mitochondrial respiratory chain (RC) dysfunction and/or decreased antioxidant mechanisms [1], [2], [3]. To counteract oxidative stress, mammalian cells are equipped with elaborate antioxidant mechanisms. Increased production of reactive oxygen species (ROS) by mitochondria can result in a vicious cycle, in which damaged mitochondria produce progressively increased amounts of ROS, leading in turn to progressive augmentation of cellular damage [4], [5]. In response to increased ROS production, cells induce the expression of a series of antioxidant enzymes, including the enzymes involved in the synthesis of glutathione: the glutamate cysteine ligase (GCL), and the glutathione synthetase (GS). GCL is a heterodimer composed of a catalytic subunit (GCLC) and a modulatory subunit (GCLM) [6], [7]. This antioxidant adaptive response is mediated by several transcriptional pathways, including NF-E2-related factor-2 (Nrf2) [8]. Under basal condition, Nrf2 is sequestered in the cytoplasm by a chaperone molecule, Keap1. Upon oxidant stimulation, Nrf2 dissociates from Keap1 and translocates into the nucleus to transactivate transcription of target genes, such as NQO1 (NAD(P)H (quinone acceptor) oxydoreductase 1) [6]. Currently, it is generally accepted that free radicals play a primary role in the aging process, especially in the tissues in which the generation of free radicals is more pronounced, such as skeletal muscle [9]. Dysregulation of Nrf2-Keap1 signaling has been described in human skeletal muscle of sedentary old adults [10]. Furthermore, aged skeletal muscle has impaired capacities of regeneration [11], [12], related to a reduced number and vitality of muscle stem cells [13]. It has been recently shown that the regulation of oxidative stress is required for self-renewal of haematopoietic stem cells [14] and neural precursor cells [15]. It has also been demonstrated that oxidative stress also modulates skeletal muscle regeneration [16], [17]. Indeed, adult stem cells from p66^ShcA^ knock out skeletal muscles displayed *in vitro* reduced levels of oxidative stress and higher proliferation rate than wild-type ones. The adult skeletal muscle stem cell pool was not impaired *in vivo* in the p66^ShcA^ mouse model while p66^ShcA^ knockout mice regenerated faster [16], showing that p66^ShcA^ and oxidative stress play an important role in skeletal muscle regeneration. However, the precise effect of increased oxidative stress on local stem cell pools in vivo is not still known in skeletal muscle, nor it is known whether such mechanisms could play a causal role in the reduction of stem cell self-renewal described in aging muscles and muscular disorders.

Skeletal muscle precursor cells or satellite cells are the myogenic stem cells responsible for post-natal growth, regeneration and repair of injured skeletal muscle [18]. Located between the basal lamina and the plasma membrane of muscle fibers, these cells are activated in response to injury and undergo cell division. Their progeny, muscle precursor cells exit the cell cycle and fuse to form new myofibers or fuse with existing myofibers [18]. Mammalian adult skeletal muscle fibers comprise four major fiber types, including slow or type I and three subtypes of fast or type II fibers, type IIa, IIx, and IIb. Each fiber type is defined by the presence of specific isoforms of myosin heavy chain (MyHC) and by a distinct program of gene expression [19]. Fiber type specification is in part dictated by an early diversification of myoblast lineages during embryonic development and is later modulated by neural and hormonal influences [20]. Given the well-characterized contribution of muscle progenitor cells to myofiber genesis, fiber specification and renewal, skeletal muscle represents a valuable model to study whether oxidative stress can affect stem cell number and function, and what factors play germane roles in such process.

Apoptosis Inducing Factor (AIF) or programmed cell death 8 (Pdcd8), is a highly conserved flavoprotein with pyridine nucleotide-disulphide oxidoreductase and DNA binding domains [21], [22]. The AIF precursor is synthesized in the cytosol and imported into mitochondria, where AIF localizes in the mitochondrial intermembrane space. Changes in mitochondrial permeability, secondary to loss of the mitochondrial membrane potential (ΔΨm) induce translocation of AIF into the cytosol and nucleus, where it may participate in chromatinolysis [23]. We and others have demonstrated an additional role for AIF as a neuronal and cardiac antioxidant, using the harlequin (*Hq*) mutant mouse, harboring a proviral insertion in the first intron of the aif gene resulting in>80% decrement in AIF expression. The *aif* hypomorphic *Hq* mutant mouse displays spontaneous progressive degeneration of cerebellar and retinal neurons [24]. Recently, it has been demonstrated that AIF deficiency does not trigger dopaminergic *Hq* neurons to degenerate but rather sensitizes them to exogenous parkinsonian mitochondrial neurotoxins through ROS-mediated toxicity [25]. *Hq* mice are also more susceptible to develop cardiac damage after acute ischemia/reperfusion and heart failure after pressure overload [26]. Mice with Cre-LoxP-mediated targeted deletion of AIF in striated muscle develop cardiomyopathy and skeletal muscle atrophy associated with defects in the mitochondrial respiratory chain and oxidative stress [27], [28], supporting a mitochondrial protective role for AIF.

We examined skeletal muscle from the *aif* hypomorphic mice in detail. Adult AIF-deficient skeletal myofibers develop atrophy with a loss of myonuclei and associated with a fast to slow fiber type switch, both in “slow” muscles such as soleus, as well as in “fast” muscles such as extensor digitorum longus (EDL). This fiber type switch was conserved in regenerated soleus and EDL muscles of *Hq* mice subjected to cardiotoxin injection. Muscle regeneration in *Hq* mice was severely delayed. *Hq* muscles displayed a decreased satellite cell pool, which could be rescued by pretreating *aif* hypomorphic mice with the manganese-salen free radical scavenger EUK-8. Primary cultures of *Hq* satellite cells suggested a longer activation step compared to the activation of WT satellite cells. However, AIF deficiency did not affect satellite cell derived mpc proliferation and differentiation. *Hq* myotubes displayed a normal size and their number of nuclei per myofiber was normal in culture. Thus, AIF protects skeletal muscles against oxidative stress-induced damage probably by protecting satellite cells against oxidative stress and maintaining skeletal muscle stem cell number and activation.

## Materials and Methods

### Animals

Wild type male mice on a B6CBACa-*A^w−J^/A* (B6CBA) background and male mice hemizygous for the X-linked harlequin mutation (*Pdcd8^Hq^; Hq*) on the same background were used. MEF2 sensor mice [29] were generously provided by Eric N. Olson (Dallas, USA). All protocols were performed according to institutional guidelines and approved by local Animal Care and Use Committees.

### Muscle injury

Animals were anesthetized. The skin was incised and 10^−5^ M cardiotoxin *Naja mossambica nigricollis* venom (Latoxan; Valence, France) was injected into soleus or EDL muscle. To follow the regeneration process, soleus and EDL muscles were collected 3, 5, 10 and 20 days post-injection.

#### Antioxidant treatment

4-week old mice were randomized to receive EUK-8 (intraperitoneal injections, three times a week, 25 mg.kg^−1^.d^−1^, Calbiochem) or an equal volume of vehicle (PBS), for the duration of the study (4 weeks).

### Antibodies

Antibodies used were: Pax7 (monoclonal, 1/5), sarcomeric MyHC (MF20, monoclonal, 1/100), fast type IIa MyHC (A4.74, monoclonal, 1/20), fast type IIb MyHC (BF.F3, monoclonal, 1/20), embryonic MyHC (F1.652, monoclonal, 1/20), and neonatal MyHC (N3.36, monoclonal, 1/20) antibodies from Developmental Studies Hybridoma Bank (Iowa University); Nrf2 (rabbit polyclonal, 1/2000) from Abcam; laminin (polyclonal, 1/200) from Sigma; cleaved caspase-3 (polyclonal, 1/20) and GAPDH (monoclonal, 1/5000) from Millipore; M-cadherin (monoclonal, 1/100) from NanoTools; LC3 (polyclonal, 1/100) from MBL; slow type I MyHC (NCL-MHCS, monoclonal, 1/20), all fast type II MyHC (NCL-MHCf, monoclonal, 1/20) from Novocastra; BrdU (monoclonal, 1/200) and MEF2D (monoclonal, 1/2500) antibodies from BD Biosciences; MyoD (polyclonal, 1/50), myogenin (rabbit polyclonal, 1/200), MEF-2 (rabbit polyclonal, 1/1000) and MEF2C (goat polyclonal, 1/500) antibodies from Santa Cruz. Secondary antibodies used were AlexaFluor anti-mouse 488 (1/200, Molecular probes), anti-rabbit Cy-2, anti-mouse Cy3 (1/250, Jackson ImmunoResearch).

### Histological analysis and immunofluorescence

For structural analysis, muscles were fixed with 4% paraformaldehyde and embedded in paraffin. Sections (6 µm) were cut and stained with hematoxylin and eosin, or incubated with antibodies against 8OHdG (7.5 µg/µL Oxis). Envision+ kit (DAKO) was used as a secondary reagent. Necrotic myofibers were visualized by Alizarin red S method. Stainings were developed using DAB (brown precipitate), slides counterstained with hematoxylin, and visualized using a Nikon Eclipse E600 microscope.

For cryostat sections, unfixed soleus and EDL were directly frozen in cold isopentane and 10 µm transverse cryostat sections were realized and incubated with the appropriate antibodies. For Pax7, M-cadherin and cleaved caspase-3 stainings, sections were unfixed, treated with PBS/Triton X−100 0.1% for 5 minutes, then incubated 30 minutes in 10% goat serum and overnight with the antibody, both antibodies were diluted in PBS/Triton X−100 0.5%. For LC3 staining, muscle sections were fixed 20 minutes at 4°C in 4% paraformaldehyde and immersed in blocking solution containing 10% goat serum, prior incubation overnight at 4°C with the primary antibody diluted in 2%BSA/PBS. TUNEL staining was performed on cryosections as described previously [30] using a TMR red TUNEL kit (Roche).

For muscle typology, fresh-frozen sections of each muscle were fixed with acetone and incubated with mouse antibodies raised against myosin heavy chains (MyHC). The percentage of type IIx myofiber was determined as the difference between the total number of type II fibres and the sum of type IIa and type IIb fibres [IIx = II− (IIb+IIa)].

After incubation with secondary antibodies and washing with PBS-T, sections were mounted in Vectashield mounting Medium (Vector laboratories).

### Isolation and purification of total RNA and real-time PCR

Primers were designed to detect transcripts for *gclm*, 5'-GCCACCAGATTTGACTGCCTTTG, 5’-TGCTCTTCACGATGACCGAGTACC-3'; *nqo1*, 5'-GCGAGAAGAGCCCTGATTGTACTG, 5'-TCTCAAACCAGCCTTTCGAATGG; *26S*, 5’-AGGAGAAACAACGGTCGTGCCAAAA, 5’-GCGCAAGCAGGTCTGAATCGTG. Total RNA was isolated using TRIzol reagent (Invitrogen). One µg of RNA was used as template for Superscript reverse transcriptase II (Promega). For realtime RT-PCR, the ABI Prism 7700 (Applied Biosystems) and SYBR Green (Applied Biosystems) were used as described in detail previously [31].

### Neuromuscular junction staining

Visualization of endplate Ach receptor distribution was performed using rhodaminated α-bungarotoxin. Receptors were incubated 30 minutes in sterile PBS containing 4 mg/mL rhodaminated α-bungarotoxin and 30 mg/mL BSA. The muscle was then rinsed in PBS and digital images of the endplates staining were obtained with fluorescence optics (Nikon eclipse E 800).

### β-Galactosidase staining of skeletal muscle

Dissected muscles from Hq and WT male mice crossed with MEF2 indicator mice (3xMEF2-lacZ) were fixed in 2% paraformaldehyde−0.2% glutaraldehyde in PBS for 30 minutes on ice. After fixation, muscles were washed 20 minutes on ice with PBS and stained overnight at room temperature in X−gal (5-bromo-4-chloro-3-indolyl-β-D-galactopyranoside) solution containing 5 mM ferrocyanide, 5 mM ferricyanide, 2 mM MgCl_2_ and 1 mg/mL X-gal. After 20 minutes wash on ice in PBS, samples were fixed overnight at room temperature with 10% formaldehyde.

### Single-fiber preparations and cell cultures

Myofibers were prepared as described previously [32] with some modifications. Briefly, soleus and EDL muscles were dissected from 8–10 week-old *Hq* or wild-type mice and digested with collagenase type I (Sigma) for one hour to yield single intact fibers that were fixed in 4% paraformaldehyde for satellite cell staining.

For clonal and differentiation analyses, single fibers were isolated from 3–4 week-old *Hq* or wild type mice as previously described [33]. In brief, muscles were partly digested in four sequential 10-min incubations in DMEM/HamF12 medium containing 0.14% pronase (Sigma). Supernatants were pooled and filtered through a 100µm cell strainer. Cells were centrifuged, washed twice and counted. Cells were grown in complete medium composed of DMEM/HamF12 (Gibco), 2% Ultroser G (Pall), 20% fetal calf serum (Gibco), penicillin, streptomycin and L-glutamine.

For clonal assays, cells were plated at low density (250 cells/ cm^2^) on poly-L-lysine coated dishes to restrict cell migration and cultured for 72–96 h, giving rise to small single-cell derived colonies of 1–20 cells. Cultures were fixed with cold methanol and processed for immunocytochemistry. For BrdU labeling, two hours before staining, cells were incubated with BrdU at a concentration of 2×10^−7^ M.

For differentiation assays, cells were plated at low density (100 cells/cm^2^) on 12-well plates coated with gelatin (type A from pig skin; Sigma). After one week, wells containing myoblasts without contaminating fibroblasts were trypsinized, pooled and expanded. Complete medium was changed every 2 days, and cultures were trypsinized before subconfluence to avoid differentiation. Two weeks later, cells were plated on gelatin coated dishes at high density (18000 cells/cm^2^). After 6 h, cells were switched to DMEM/HamF12 containing 2% horse serum. Cultures were fixed 72 h later in 4% paraformaldehyde and processed for immunocytochemistry.

### Immunocytochemistry

Fixed myofibers were permeabilized with 0.2% Triton X–100 for 10 minutes and collected by centrifugation. Fibers were washed with PBS, blocked with 20% goat serum in PBS for 1 h and incubated with mouse monoclonal anti-Pax7 diluted in 0.35% λ-Carrageennan (Sigma) overnight at 4°C, then with secondary antibody. Nuclei were stained with To-Pro-3 and myofibers mounted. The percentage of satellite cells was determined by counting the Pax7-positive nuclei among at least 1 000 nuclei in six different cultures of each condition.

Clones on poly-L-lysine were gently washed with serum-free prewarmed DMEM and immediately fixed in cold methanol for 5–10 minutes. Cells were then treated with 4N HCl for 20 minutes at room temperature, rinsed with 0.1 M sodium tetraborate and stained with monoclonal anti-BrdU and polyclonal anti-MyoD antibodies and Cy3- and Cy2-conjugated secondary antibodies. Cells were stained with To-Pro-3 and mounted. The percentage incorporation of BrdU was determined by counting all BrdU-positive cells among the MyoD-positive cells in three different cultures for each condition.

To analyze the differentiation potential of satellite cell derived myoblasts, differentiating cells were permeabilized with 0.2% triton X−100 for 10 minutes, incubated with mouse anti-sarcomeric MyHC and rabbit anti-myogenin antibodies and Cy3- and Cy2-conjugated secondary antibodies. Cells were stained with To-Pro-3 and mounted. The diameters of at least 400 myotubes from three different primary cultures were measured in a region where myonuclei were absent and diameter was constant. The number of nuclei per cell was counted in at least 400 myotubes per culture.

### Western blot analysis

Muscles were frozen and total protein extracts were prepared by using RIPA lysis buffer plus protease inhibitors, as described in detail previously [31]. 40µg of each protein lysate were separated on 10% SDS-PAGE, transferred to a PVDF membrane, and probed with the indicated antibodies prior the corresponding horseradish peroxidase (HRP)-conjugated secondary antibodies (1/2000, Jackson ImmunoResearch) and ECL detection.

### Statistical Analysis

The results are presented as means ± SEM. Statistical analyses were performed using INSTAT 3.0 software (GraphPad, San Diego) and Student’s t-test or ANOVA followed by Tukey’s post-test when appropriate. Statistical significance was accepted at a P value<0.05.

## Results

### Atrophy and oxidative stress in AIF-deficient skeletal muscle

**Figure 1:**
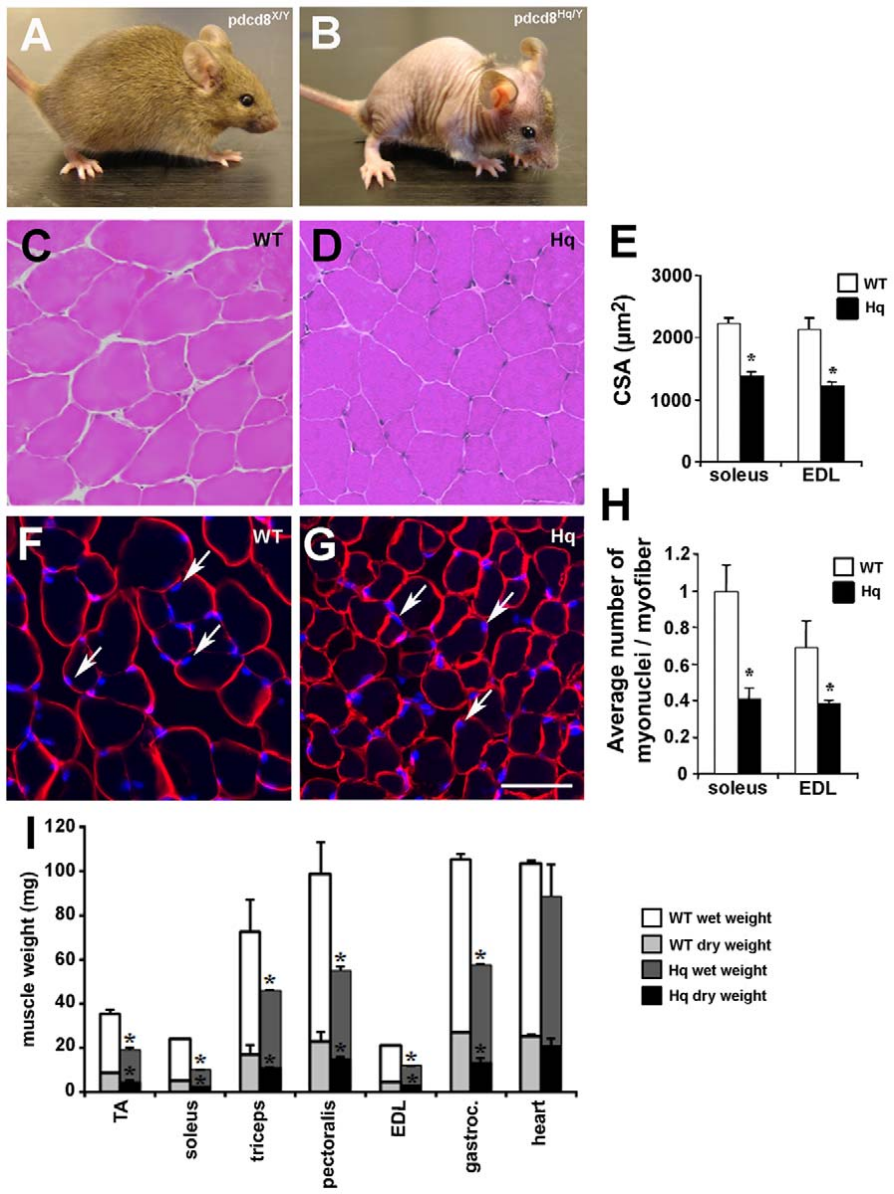
Atrophy in *Hq* skeletal muscle. Six-week old, AIF hypomorphic, harlequin (*Hq*) mutant mice (pdcd8*^Hq^* ^/Y^; B) show reduced body size, lordokyphosis and reduced hair density in comparison with wild-type littermate (WT; A). The hematoxylin-eosin staining (C, D) on transverse sections of 3 month-old WT (C) and *Hq* (D) soleus muscles show atrophy in *Hq* mice. Bar = 20 µm. Quantitation of fiber cross sectional area (CSA) (E) confirms the atrophy in *Hq* soleus and EDL muscles of 3-month old mice of indicated genotype. Sections of WT (F) and *Hq* (G) EDL (F, G) muscles were immunostained using anti-laminin and bis-benzimide to reveal sarcolemma and nuclei. Arrows in F and G show myonuclei inside the sarcolemma. The number of myonuclei is reduced in *Hq* soleus and EDL muscles, as quantitated in H. (I) Dry and wet weights of individual skeletal muscles and cardiac muscle of 2 month-old *Hq* and WT mice confirm a muscle weight loss in *Hq* mice. gastroc.: gastrocnemius.Bar = 25 µm. * indicates P<0.05 vs WT.

At birth, Harlequin (*Hq*) mice are visibly smaller than their wild type littermates (not shown). At 6 weeks, mutant mice are lean and display signs of alopecia (Fig. 1A, B) [28]. At 2 months of age, all of the analyzed skeletal muscle wet weights were severely decreased in mutants compared to littermate control mice (Fig. 1I). Considering the metabolic role of AIF, we measured the dry weight of some skeletal muscles and the cardiac tissue by subjecting the tissues to drying at 105°C for 24 hours. All dry weights corresponded systematically to approximately 24% of the wet weight of each analyzed muscle, in *Hq* as in wild type samples (Fig. 1I). Reduced dry and wet muscle mass could be derived from either the reduction in myofiber number, or a reduction in myofiber size, or both. Histological analysis revealed that myofibers from both soleus and EDL muscles were clearly atrophied (Fig. 1C–G) in 12 week-old *Hq* mutant mice. Indeed, fiber cross sectional area was on average 40% smaller in “slow twitch” soleus muscle as well as in the “fast twitch” EDL from *Hq* mice compared to WT EDL and soleus muscles, indicating that *Hq* skeletal muscle atrophy is muscle type independent (Fig. 1E). The number of fibers per soleus or EDL muscle was similar in adult WT and *Hq* mice (Table 1), suggesting that the reduced muscle mass observed in *Hq* mice is mainly due to atrophy and not hypoplasia. In several experimental models such as denervation and mechanical muscle unloading, skeletal muscle atrophy is associated with loss of nuclei per myofiber (reviewed in [34]). We therefore counted the number of nuclei under the basal lamina and showed that the number of myonuclei per myofiber cross-section is significantly decreased in both soleus and EDL *Hq* muscles compared to wild-type ones in two month old mice (Fig. 1F–H).

**Table 1:** Absolute number of myotubes in regenerating muscles and myofibers in adult muscles.

	Soleus		EDL	
	WT	Hq	WT	Hq
**3 days P-I**	264+/−22	3+/−2	3+/−3	0+/−0
**5 days P-I**	451+/−52	6+/−3	448+/−43	69+/−45
**10 days P-I**	567+/−96	113+/−14	723+/−96	645+/−66
**20 days P-I**	656+/−64	317+/−54	800+/−56	728+/−75
**adult**	731+/−96	672+/−85	897+/−135	930+/−128

AIF was initially characterized as a caspase-independent death effector [35]. So we investigated cell death on soleus and EDL sections of 2 month-old wild-type and *Hq* mice. To label cells undergoing apoptosis, we used an antibody to the activated form of caspase-3 (Fig. S1C, S1D). No activated caspase-3 labeled cell was detected in soleus and EDL muscles of both wild-type and *Hq* mice, with the exception of one cell from all analyzed sections. The same observation was obtained by TUNEL staining (data not shown). Autophagic cells were detected by immunohistochemistry using an antibody against the microtubule-associated protein light chain 3 (LC3), protein essential for the autophagosome membrane formation (Fig. S1A, S1B). Rare autophagic cells were detected in both wild-type and *Hq* muscle sections (Fig. S1A). Next, we stained sections with the Alizarin method, which highlights calcium deposition that occurs in necrotic fibers. Necrotic fibers were observed in soleus and EDL muscles of wild-type and *Hq* mice, 3 days after a local cardiotoxin injection. However, adult soleus and EDL muscles of both genotypes did not display any necrotic fibers (Fig. S1E). Taken together, these data show that *Hq* muscles present an atrophic phenotype associated with a loss of myonuclei, without any obvious sign of apoptosis, autophagy or necrosis.

**Figure 2:**
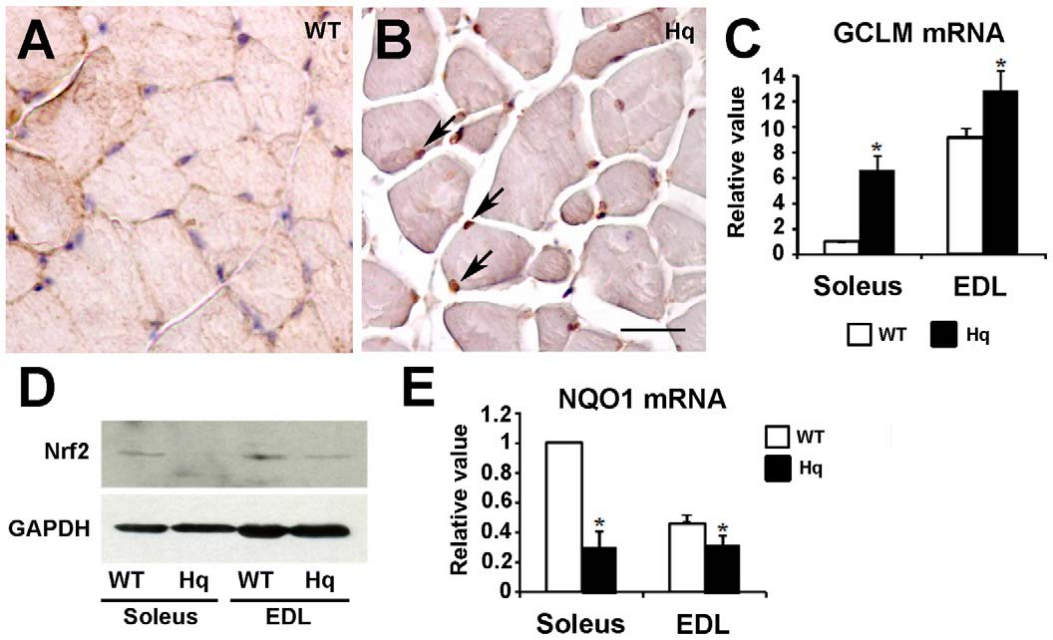
Oxydative stress in Hq skeletal muscle. The 8OHdG immunostaining (A, B) on transverse sections of 3 month-old WT (A) and *Hq* (B) soleus muscles show increased free radical damage in *Hq* mice. Bar = 10 µm. (C) Real-time PCR analysis of *gclm* transcript abundance, gene overexpressed in oxidative stress models in soleus and EDL from 2-month old wild-type and *Hq* mice, Western blot (D) and real-time PCR (E) analyses of endogenous Nrf2 (D) and NQO1 (E) reveal dysfunctions in the Nrf2 redox signaling.

It has previously been proposed that AIF directly or indirectly regulates free radical scavenging. In cerebellar and retinal neurons and in cardiomyocytes, AIF enhances cellular survival via its ability to protect tissues against oxidative stress-induced cell death [24], [26]. To evaluate free radical damage in skeletal muscle cells, we stained soleus and EDL muscles from 3 month-old *Hq* and WT mice for 8OHdG, a marker for DNA damage (Fig. 2A–B). In WT soleus and EDL muscle, cells were weakly stained for 8OHdG. In contrast, nuclei were strongly labeled with the 8OHdG antibody in both the “slow” soleus muscle and in the “fast” EDL muscle of *Hq* mice. DNA damage in *Hq* muscle cells was associated with increased expression of GCLM, the modulatory subunit of GCL (Fig. 2C), gene overexpressed in several oxidative stress models [36], which strongly suggest increased ROS production in Hq muscles. In contrast, *Hq* soleus and EDL muscles displayed a dramatic reduction of Nrf2 protein accumulation (Fig. 2D), correlated with a decrease in transcript amounts of its downstream target gene, *Nqo1* (Fig. 2E). These dysfunctions in the Nrf2 redox signaling could potentially participate in increasing oxidative stress in *Hq* tissues.

Conclusively, AIF-deficient skeletal muscles display characteristics of muscular myopathy and aging, such as atrophy and increased oxidative stress-induced damage in adult myofibers.

### AIF-deficiency induces a slow fiber type switch

**Figure 3:**
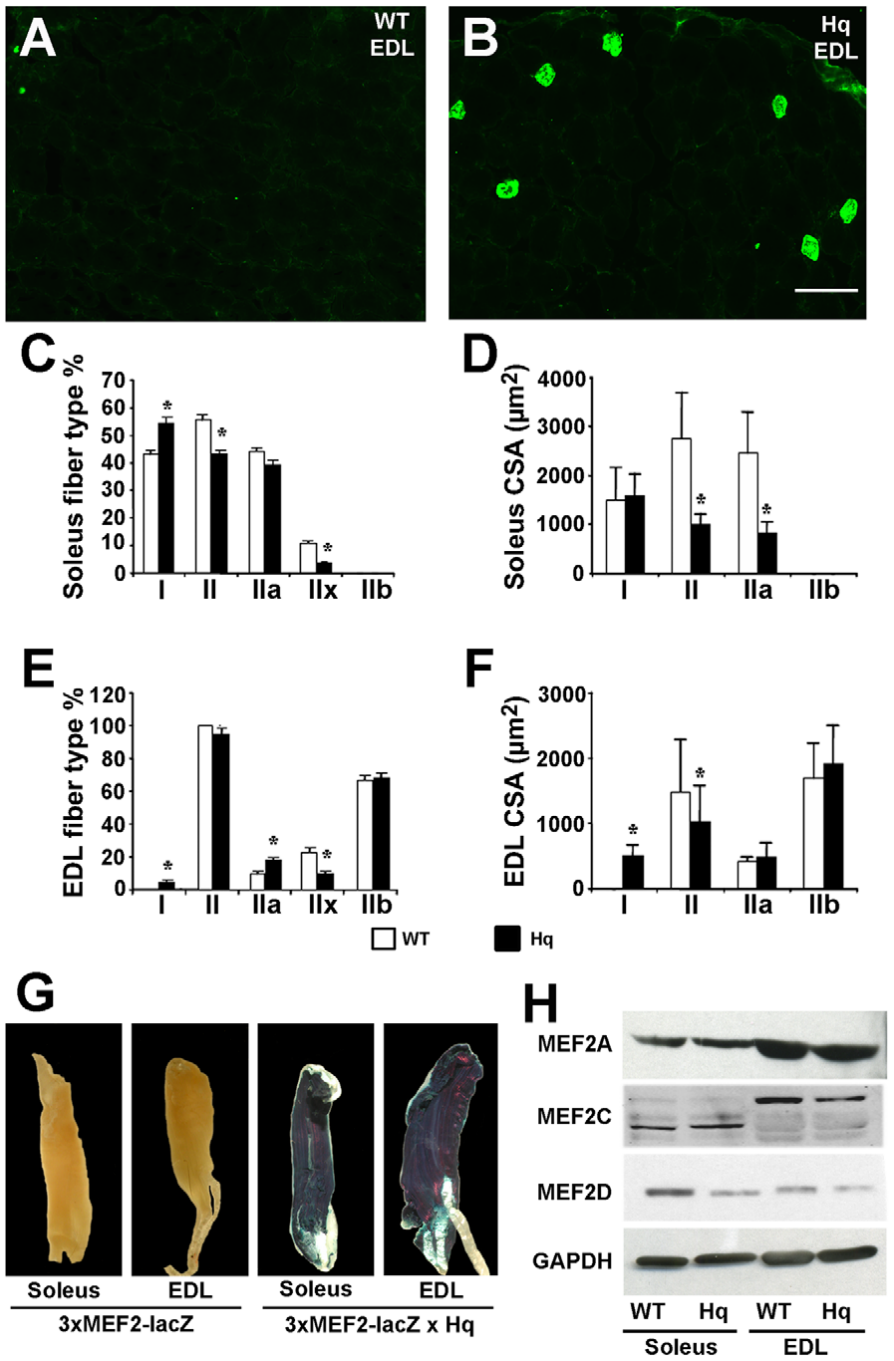
AIF deficiency induces a slow fiber type switch, associated to an increased MEF2 activity. Immunofluorescent analysis of type I myofibers on transverse sections of WT EDL muscle (A) and *Hq* EDL muscle (B). Bar = 60 µm. Quantification of type I and type II fibers in WT and *Hq* soleus (C) and EDL (E) muscles confirms the slow fiber type switch in *Hq* mice. Quantitation of fiber cross sectional area (CSA) specifies the atrophy of some fiber types in soleus (D) and EDL (F) muscles. * indicates P<0.05 vs WT. (G) β-Galactosidase staining of soleus and EDL muscles isolated from 2-month-old MEF2 indicator mice (3×MEF2-lacZ) or *Hq* mice crossed with MEF2 indicator mice (3×MEF2-lacZ×Hq). Expression of the *lacZ* transgene depended on MEF2 activity. Blue staining indicates augmented MEF2 activity in tissues. Up-regulation of desMEF2-lacZ transgene expression in 2 month-old *Hq* soleus and EDL muscles occurs without any significant changes in the abundance of major MEF2 isoforms (H). We notice that MEF2C is differentially spliced in soleus and EDL muscles.

To further characterize the muscle abnormalities of *Hq* mutant mice, we analyzed the typology of soleus and EDL muscles in 3 month-old *Hq* and WT littermates by immunofluorescence. In mice, the soleus is a mixed muscle with approximately the same number of both slow-type I and fast-type IIa fibers [37] (Fig. 3C). In Harlequin soleus muscle, type I fibers increased significantly compared to WT soleus muscle, whereas the percentage of all type II fibers decreased significantly in *Hq* mice. Both type IIa and IIx fibers in soleus muscle were less abundant in *Hq* compared to WT soleus muscle. EDL is exclusively composed of type II fibers in mice [37] (Fig. 3A, 3B, 3E). Interestingly, *Hq* EDL was composed of 4.5±0.5% of type I fibers, with corresponding less type II fibers, more specifically significant decrease of type IIx fibers. Indeed the amount of type IIb fibers, the fastest one, were not modified, whereas type IIa fibers, the slowest among the type II fibers, were more abundant in *Hq* EDL muscle compared to WT control mice. No central nuclei were observed in *Hq* myofibers (Fig. 1G). Furthermore no embryonic or neonatal MyHC expression was detected in adult *Hq* and WT soleus and EDL muscles by immunohistochemistry (data not shown), indicating that fibers in *Hq* and WT mice were not immature regenerating fibers. Combined, the typological analysis indicates that AIF-deficient muscles present a fiber type switch from fast to slow fibers, both in “slow” muscles such as soleus muscle, as well as in “fast” muscles such as EDL.

The analysis of the cross sectional area of fibers expressing each type of MyHC indicated a specific response to the atrophy for each fiber type. Fibers expressing type II MyHC and in particular type IIa were indeed the only fiber type affected by atrophy in *Hq* soleus muscles (Fig. 3D). In *Hq* EDL, nor type IIa, or type IIb fibers showed any decrease in their cross sectional area (Fig. 3F). However, fibers expressing type II MyHC are globally atrophic in *Hq* EDL, suggesting that type IIx fibers are certainly responsible for this atrophy in this *Hq* muscle, in concomitance with the appearance of type I fibers characterized by a small cross section area (Fig. 3F).

Fiber type specification is partly dictated by an early diversification of myoblast lineages during embryonic development and is subsequently modulated by neural and hormonal influences [20]. As muscle innervation is a major determinant of the muscle fiber phenotype, and since *Hq* mice were earlier characterized to display progressive degeneration of retinal and cerebellar neurons, we wanted to exclude that the observed muscle defects were due to altered neurohumoral input, by analyzing the distribution of Acetylcholin Receptor (AchR) in neuromuscular junctions of *Hq* and WT soleus and EDL muscles (Fig. S2A–D). Neuromuscular junction structure appeared similar in *Hq* and WT muscles. No fragmentation, no defect in their maturation was evident in *Hq* and WT soleus and EDL, suggesting that neurotransmission is not altered in *Hq* mice, and that the specific muscle defects were cell autonomous.

To determine whether the fiber switch observed in *Hq* transgenic mice was linked to MEF2 activation in vivo, we bred *Hq* and wild-type mice with MEF2 indicator transgenic mice harboring a *lacZ* transgene linked to three copies of the MEF2 consensus binding site from the *desmin* promoter (3×MEF2-lacZ) [38]. In 3×MEF2-lacZ mice, expression of *lacZ* depends on MEF2 transcriptional activity [39]. LacZ expression in soleus and EDL muscles was detected by β-galactosidase staining (Fig. 3G). To be able to compare the levels of lacZ expression, the β-galactosidase reaction was realized in a limited period of time. In these conditions, *lacZ* expression was barely detectable in wild-type soleus and EDL muscles. However, β-galactosidase staining was highly strong in *Hq* soleus and EDL muscles, indicating a high MEF2 activity in both soleus and EDL muscles of Harlequin mice, without any significant changes in expression of major MEF2 isoforms (Fig. 3H). We notice that the major MEF2C variant is different in soleus and in EDL muscles (Fig. 3H). As MEF2 activity promotes slow and oxidative myofibers (type I and IIa fibers) [39], [40], the fiber switch from fast to slow fibers observed in soleus and EDL muscles of Harlequin mice probably results from an increase of MEF2 activity in these skeletal muscles.

### AIF is essential for skeletal muscle regeneration

Oxidative stress is known to play an important role in muscle aging [9]. Loss of muscle mass, or sarcopenia, observed in aging muscles, is directly related to a significant reduction of the regenerative potential of muscles. As *Hq* skeletal muscle fibers display signs of oxidative stress, we analyzed the regenerative capacity of soleus and EDL from WT and mutant mice. A single injection of cardiotoxin causes an almost complete degeneration of the myofibers within 24 h, followed by proliferation of myoblasts, with evidence of regenerated myotubes in the next following days [41].

**Figure 4:**
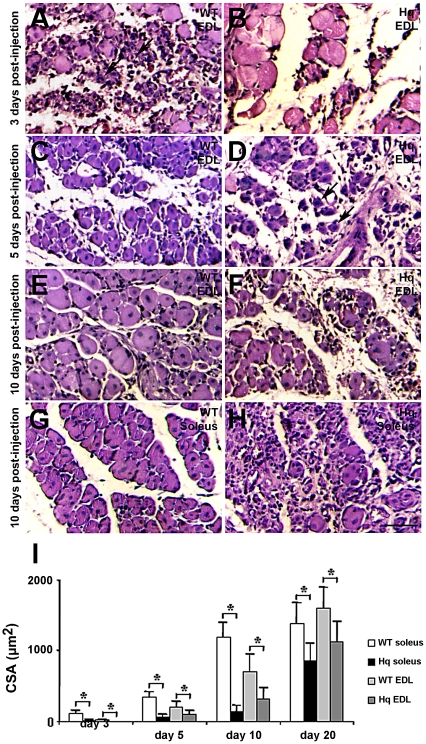
Delayed skeletal muscle regeneration in *Hq* mice. Hematoxylin-eosin histological staining of transverse sections of regenerating EDL (A–F) and soleus (G, H) muscle from WT (A, C, E and G) and *Hq* (B, D, F and H) mice 3- (A, B), 5- (C, D) and 10 days (E–H) following cardiotoxin injury. Bar = 10 µm. Arrows in A and D point out newly formed myotubes with central nuclei. (I) Quantitation of cross sectional area (CSA) of myotubes in regenerating soleus and EDL muscles 3, 5, 10 and 20 days following cardiotoxin injection confirms the delay in muscle regeneration in *Hq* mice.

Comparison of transverse sections of the regenerating muscles from *Hq* mice versus WT mice showed a delay of the differentiation process in regenerating muscles of *Hq* mutant mice (Fig. 4). At 3 days post-injection (P–I), numerous myoblasts lined up and fused in between necrotic myofibers, as well as some young forming myotubes were observed in regenerating EDL of WT mice (Fig. 4A). In contrast, most of the cells are necrotic fibers in *Hq* mice at this stage (Fig. 4B). At 5 days P–I, myotubes with central nuclei were the predominant cell type observed in regenerating EDL of WT mice (Fig. 4C). These myotubes exhibited a larger surface area and were more abundant in comparison to those of the regenerating EDL in *Hq* mice (Fig. 4D, 4I, Table 1). This size difference of the myotubes persisted in the following days as shown at 10 days P–I (Fig. 4E, 4F, 4I). This delay in the regenerative process of *Hq* EDL was also evident and more pronounced in soleus muscle than in EDL: whereas large myotubes were predominant in regenerating soleus of WT mice at 10 days P–I, only few small myotubes were detected in *Hq* mice (Fig. 4G, 4H, 4I, Table 1). Taken together, *Hq* skeletal muscle displayed a clear delay in the regenerative process of both EDL and soleus muscle compared to WT mice in response to cardiotoxin injection.

**Figure 5:**
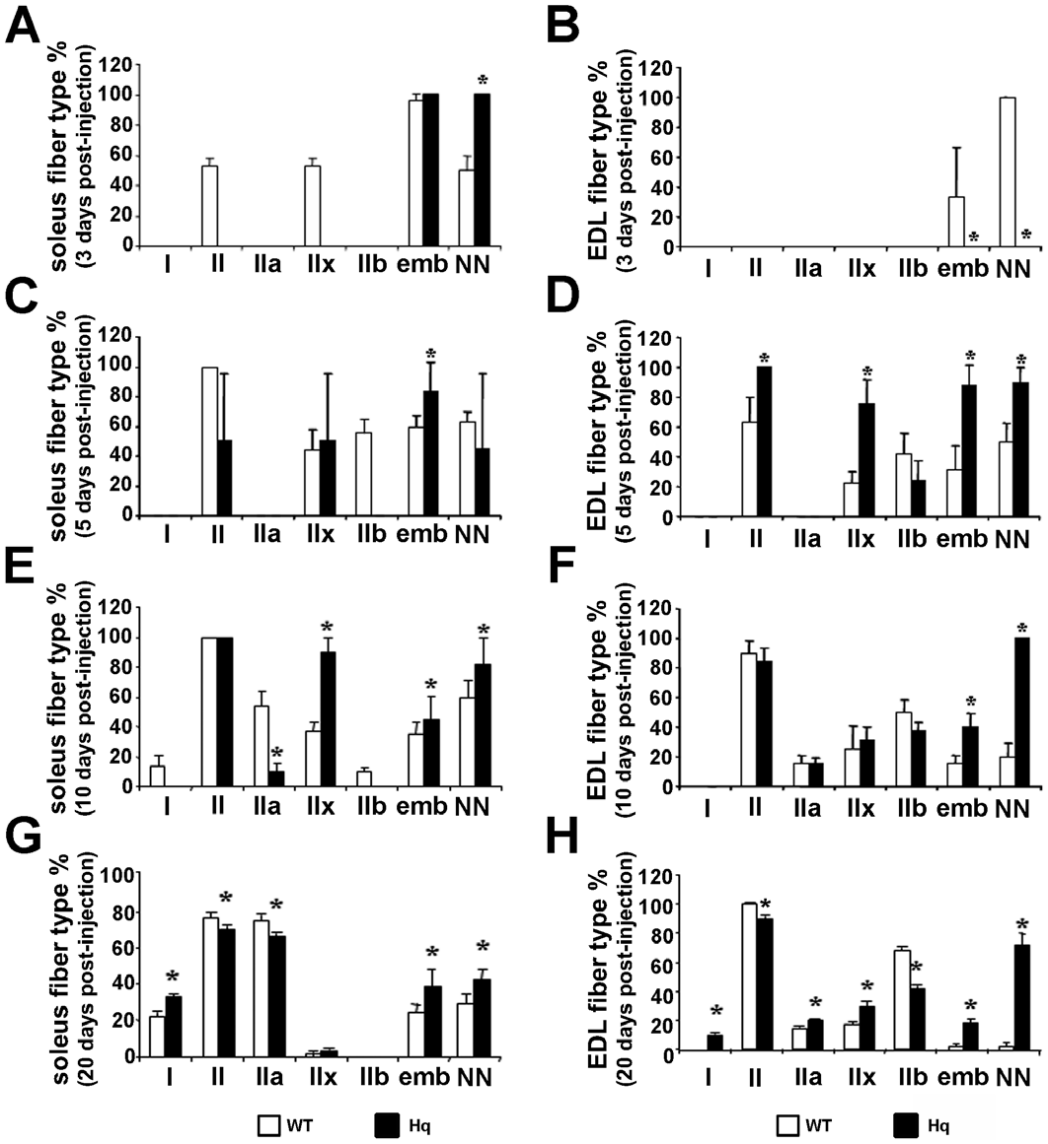
A slow fiber type switch in regenerating *Hq* muscles. Quantification of fibers expressing type I, type II, embryonic and neonatal MyHC in WT and *Hq* soleus (A, C, E, G) and EDL (B, D, F, H) muscles 3 (A, B), 5 (C, D), 10 (E, F) and 20 (G, H) days following cardiotoxin injury indicates a slow fiber type switch and an increase in immature regenerating myofibers in *Hq* mice, the latter corroborating the delay in skeletal muscle regeneration of *Hq* mice. * indicates P<0.05 vs WT.

Next we analyzed the typology of regenerating soleus and EDL muscles from 3 month-old *Hq* and WT mice by immunohistochemistry (Fig. 5). At 3 days P–I, regenerated WT soleus muscles contain embryonic MyHC expressing fibers, which coexpress the neonatal and/or type IIx MyHC isoform (Fig. 5A). At this stage, the rare myofibers observed in regenerating *Hq* soleus only express embryonic and neonatal MyHC (Fig. 5A and Table 1). Type IIx expression appears later (5 days P–I) in myofibers of *Hq* soleus muscles, as myofibers expressing type IIb MyHC are already transiently observed in WT soleus (Fig. 5C, 5E, Table 1). No type IIb MyHC expressing myofibers has been observed at any stage of regeneration in *Hq* soleus (Fig. 5C, 5E, 5G). Most myotubes are formed in *Hq* soleus from 10 to 20 days P–I, much later than in the WT soleus, which is a sign of a delayed regeneration in this muscle of *Hq* mice (Table1). The delay in the regenerative process in *Hq* soleus is also characterized by a delay in appearance of type I myofibers: 20 days P–I in *Hq* soleus vs 10 days P–I in WT soleus (Fig. 5E, 5G). Moreover, myofibers expressing embryonic and neonatal MyHC are more abundant in *Hq* soleus than in WT soleus both 10 and 20 days P–I, reflecting that *Hq* myofibers are less mature fibers than in WT mice at these stages of regeneration. At 20 days P–I, regenerated *Hq* soleus muscle exhibited a significantly higher content of type I fibers compared to WT regenerated soleus muscle (WT, 21.6±2.6%; *Hq*, 32.8±1.5%; P<0.05; Fig. 5G) and a significant decrease of total type II and specially type IIa fibers (WT, 74.8±2.5%; *Hq*, 66.1±1.4%; P<0.05; Fig. 5G). The distribution of type IIx fibers (Fig. 5G) was equal in *Hq* and WT soleus muscle.

In EDL, the regeneration process is slightly delayed in comparison to the soleus. Myotubes are rare in WT mice and non-existent in *Hq* mice 3 days after the cardiotoxin injection (Table 1). 5 days P–I, type IIx and IIb MyHC expressing myotubes are present in both WT and *Hq* EDL muscles, despite a significantly higher content of type IIx fibers in *Hq* EDL (Fig. 5D). The muscle typology of regenerating EDL in *Hq* and WT mice is similar 10 days after the cardiotoxin injection, except a higher distribution of myofibers expressing embryonic and neonatal MyHC in *Hq* EDL, which is observed from 5 to 20 days P–I (Fig. 5D, 5F, 5H). This data confirms that *Hq* EDL regeneration is delayed compared to WT muscles. Type I fibers, the “slowest” fibers in skeletal muscle, were not observed in WT animals, whereas AIF-deficiency readily induced the expression of type I MyHC in 9.7±0.8% of the newly regenerated fibers in mutant EDL muscle at 20 days P–I, similar to *Hq* non-regenerated EDL muscle (Fig. 5H, Fig. 3E). Total type II fibers were less abundant in regenerated *Hq* EDL muscle compared to regenerated WT EDL muscle (Fig. 5H). However, type IIa and IIx fibers, the “slowest” fibers among the type II fibers, were significantly more abundant in mutant muscle compared to WT muscle, with a concomitant reduction in type IIb fibers in *Hq* regenerated EDL muscle compared to WT controls (Fig. 5H).

Conclusively, the typology of *Hq* mutant muscles 20 days after the injection of cardiotoxin confirms a “fast to slow” fiber-type switch, similar to that described in non-regenerated soleus and EDL muscles of AIF-deficient mice (Fig. 3C, 3E), associated with an important distribution of immature regenerating fibers in the *Hq* muscles (Fig. 5).

### AIF regulates skeletal muscle cell precursor activation

The remarkable capacity of skeletal muscle for efficient regeneration following injury is due to the activation and proliferation of a pool of skeletal muscle precursor cells, known as satellite cells [18]. Defects in the satellite cell pool might contribute to a poor capacity of regeneration and to fiber atrophy, two phenotypes that we described in *Hq* mice (Fig. 1 and 4). It has been shown that the regulation of oxidative stress is required for self-renewal of haematopoietic stem cells, neural precursor cells [14], [15], and modulates myogenic differentiation [16], suggesting that oxidative stress could play a crucial role in stem cell self-renewal. However, it is currently unknown if the satellite cell pool is also subject to oxidative stress.

**Figure 6:**
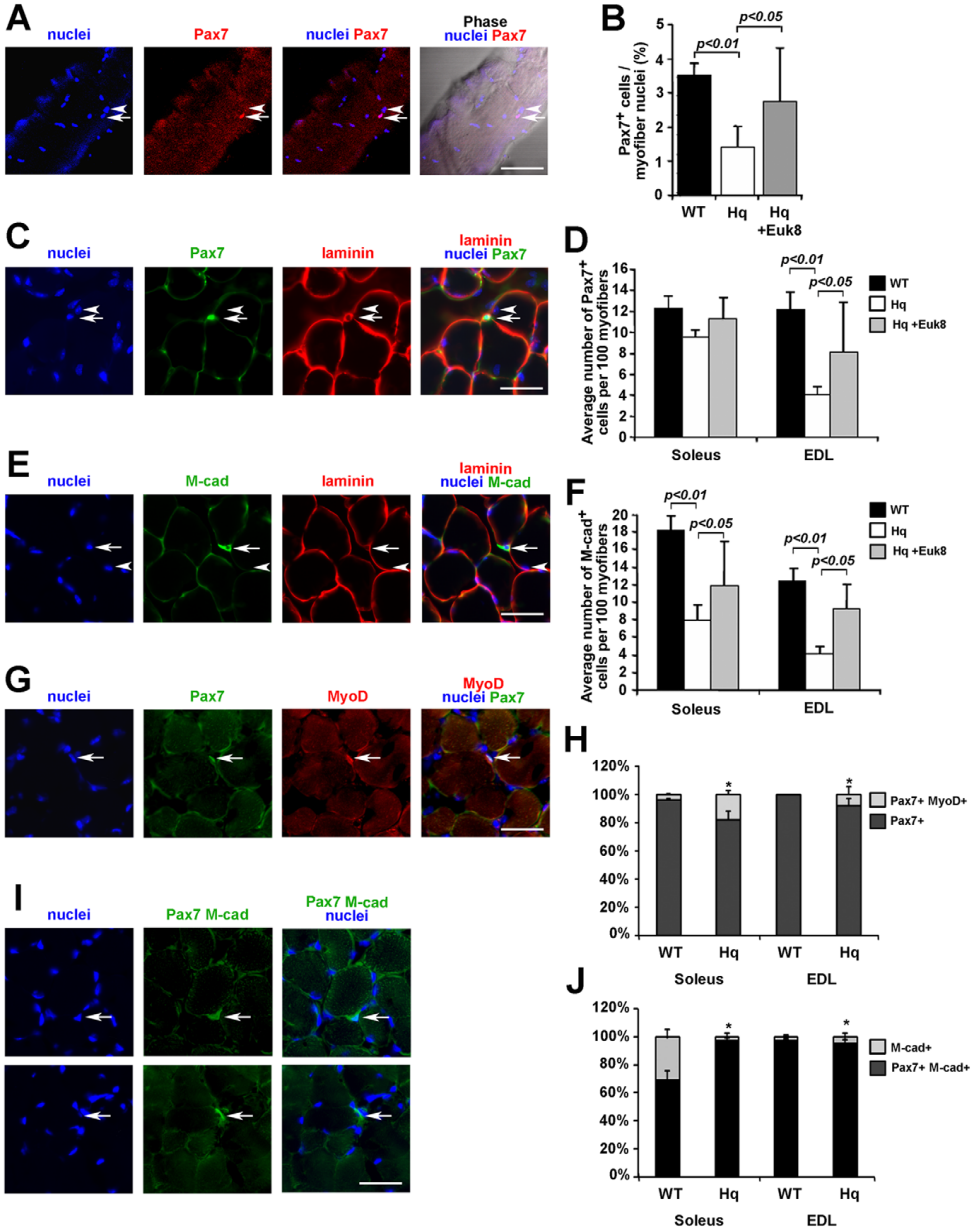
Loss of satellite cells in AIF deficient mice. (A) Representative picture of a Pax7-positive satellite cell attached to a freshly isolated myofiber from WT EDL muscle. (B) Quantification of the number of Pax7-positive satellite cell nuclei per 100 myofiber nuclei, indicates a significant reduction of Pax7-positive satellite cells in isolated *Hq* EDL myofibers. Measurements were made from six different animals of each genotype by counting the number of Pax7-positive cells per fiber and the total number of myofiber nuclei per fiber as visualized by Bis-benzimide staining. Pax7 (C) and M-cadherin (E) antibody staining of WT EDL muscles show Pax7^+^ (arrow in C), M-cadherin^+^ nuclei (arrow in E) and myonuclei (arrowheads in C and E) located under the basement membrane, marked by anti-laminin staining from WT EDL cross sections. Quantification of the number of Pax7-positive (D) and M-cadherin-positive (F) satellite cell nuclei per 100 myofiber cross sections indicates a significant reduction of Pax7 positive satellite cells in *Hq* EDL muscles and a reduction of M-cadherin^+^ cells in soleus and EDL *Hq* muscles. (G, I) Representative pictures of an activated satellite cell (arrow in G), co-immunostained with MyoD and Pax7 antibodies and of satellite cells expressing either Pax7 and M-cadherin (arrow in I, top panel) or M-cadherin only (arrow in I, lower panel) on *Hq* EDL cross sections. (H) Quantification of the number of activated satellite cells (Pax7 and MyoD positive nuclei) and of non-activated satellite cells (Pax7-positive MyoD-negative nuclei) indicated a higher amount of activated satellite cells in both *Hq* soleus and EDL muscles compared to WT muscles. (J) Among the M-cadherin-positive satellite cells, about 30% are Pax7-negative in the WT soleus only. Measurements in D, F, H and J were made from five different animals of each genotype on soleus and EDL muscle sections. Nuclei were stained with Bis-benzimide. Bar = 10 µm (A), 25 µm (C, E, G, I).

Therefore, to explore whether the activation, proliferation, and differentiation of satellite cells are prone to AIF deficiency induced oxidative stress, we isolated myofibers from EDL muscle of 2-month old WT and *Hq* mice, and from *Hq* mice treated with EUK-8, an antioxidant with powerful superoxide dismutase (SOD), catalase and oxyradical scavenging properties. The number of Pax7 positive satellite cells from freshly isolated myofibers amounted to 3.5±0.3% in WT EDL, while *Hq* EDL myofiber cultures displayed almost half the amount of Pax7 positive satellite cells per myofiber (1.4±0.6%) (Fig. 6A, 6B). After 4-week old *Hq* mice were treated with Euk-8, the number of Pax7 positive cells was significantly increased in their EDL muscle (2.7±1.5%) (Fig. 6B). These single fiber experiments indicate that *Hq* skeletal muscles display a reduction in the satellite cell pool and that this defect can be partly rescued by antioxidant treatment. To confirm these results and exclude a possible detachment of satellite cells during the myofiber isolation, we identified satellite cells on soleus and EDL sections by labeling them with a Pax7, a M-cadherin, or a combination of these antibodies. In EDL muscle, the number of Pax7 positive satellite cells per 100 myofibers was roughly 3 times less in *Hq* mice than in WT mice (4.0±0.4 Pax7 positive cells per 100 myofibers in *Hq* EDL vs 12.2±1.6 in WT EDL) (Fig. 6C, 6D). Similar results were obtained using M-cadherin labeling (Fig. 6E, 6F). In WT soleus, the number of M-cadherin positive cells per 100 myofibers was approximately 50% higher than the number of Pax7 positive cells: 18.2±0.6 M-cadherin positive cells vs 12.2±1.1 Pax7 positive cells (Fig. 6D, 6F). Double labeling with Pax7 and M-cadherin antibodies confirmed that 30% of the M-cadherin positive satellite cells seemed to be Pax7 negative in these WT soleus (Fig. 6I, 6J). In these experiments, we were not able to discriminate between Pax7/M-cadherin double labeled cells and Pax7 positive M-cadherin negative cells, since both antibodies are mouse monoclonal antibodies. In *Hq* soleus, the number of Pax7 positive cells per 100 myofibers (9.5±0.7) was similar to that of M-cadherin positive cells (7.9±1.7), suggesting that AIF deficiency leads to a depletion of M-cadherin positive satellite cells in both soleus and EDL muscles and an apparent loss of Pax7-positive cells only in the EDL muscle (see discussion). The Euk8 treatment of *Hq* mice partially rescued any loss of satellite cells observed in *Hq* EDL and soleus muscles by both analyses, using isolated myofibers and cryosections (Fig. 6). Moreover, in both soleus and EDL muscles of *Hq* mice, roughly 10 to 20% of the Pax7 positive satellite cells were in an activated status, since they co-expressed Pax7 and MyoD (Fig. 6G, 6H), while rare or no satellite cells were activated in muscle controls.

**Figure 7:**
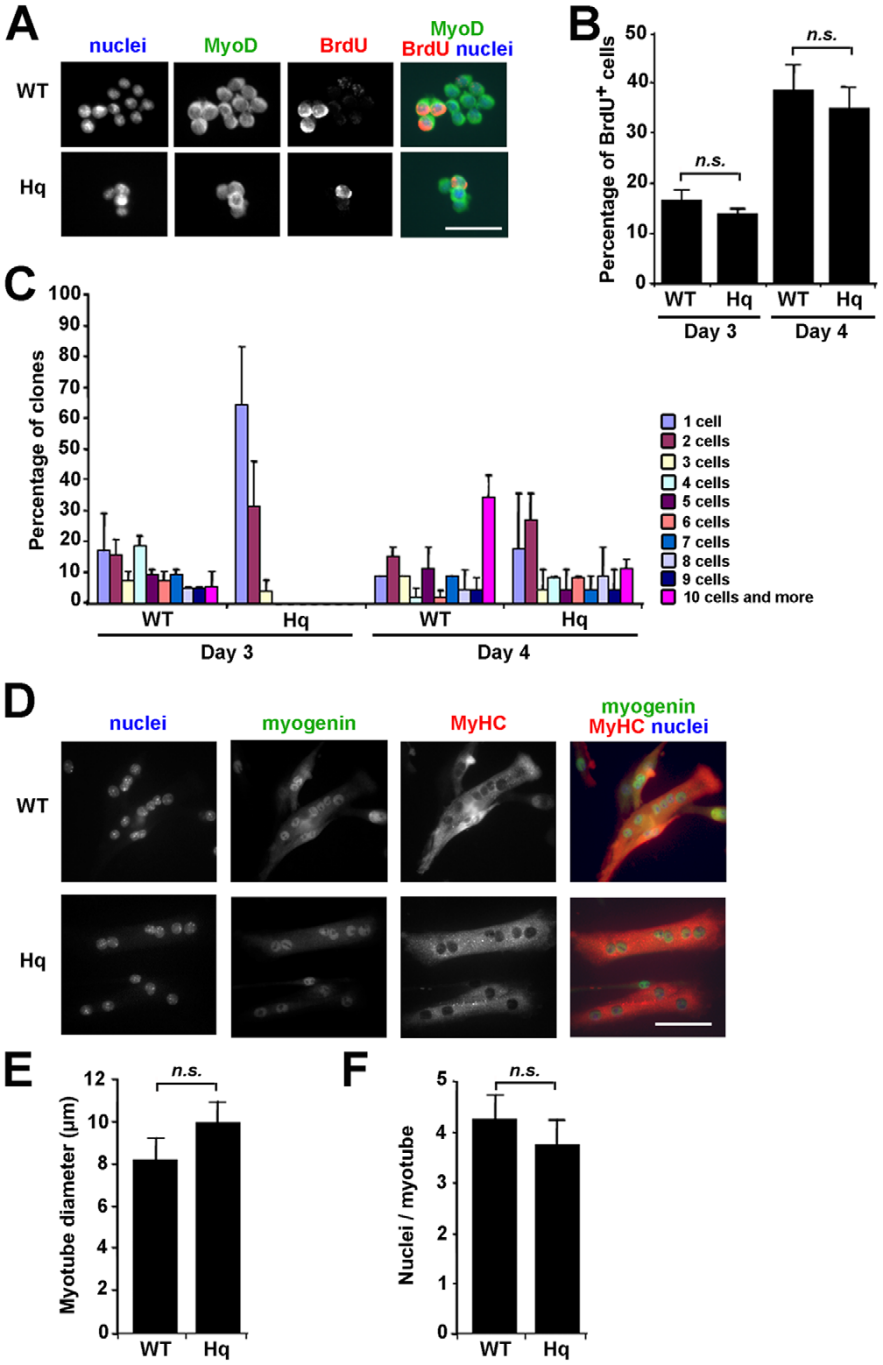
AIF deficiency decreases satellite cell clonogenicity, without affecting their proliferation and their differentiation. (A) Representative picture of WT and *Hq* microcolony grown for 4 days. Cells were stained with anti-MyoD and anti-BrdU antibodies and Bis-benzimide to reveal myogenic S-phase and total nuclei, respectively. Bar = 10 µm. (B) Quantification of the percentage of cycling cells in WT and *Hq* myogenic colonies of at least 2 cells, grown for 3 or 4 days, after 2 h BrdU pulse and stained as in A, shows no difference in the proliferation rate of *Hq* and WT mpc. (C) Assessment of WT and *Hq* microcolony formation after 3 or 4 days in culture and stained as in A, indicates a delay in the microcolony formation of *Hq* satellite cell primary cultures. (D) Differentiated WT and *Hq* myocytes plated at high density and grown for 3 days were detected by immunostaining for myogenin and sarcomeric MyHC. Total nuclei were stained with Bis-benzimide. Bar = 15 µm. (E, F) After 3 days in culture, the myotube diameter (E) and the number of nuclei per myofiber (F) were similar in WT and *Hq* primary cultures. At least 400 myotubes were analyzed. *n.s*. not significant.

To test whether activated satellite cells proliferate normally, primary cultures from hindlimb muscles of WT and *Hq* mice were plated at low density so that the number of cells per colony could be monitored three and four days after plating (Fig. 7C). Mitotic MyoD-positive progenitor cells from colonies of at least 2 cells were examined in 3 and 4 day old primary cultures after 2 h pulse of BrdU (Fig. 7A). The rate of S-phase entry in primary cultures from WT and *Hq* muscles at day 3 and 4 was similar (Fig. 7B). Conclusively, proliferation is not affected in cultured AIF-deficient mpc.

Clonogenicity, i.e. the ability to expand at a single cell level is an important feature of self-renewing stem cells. To test the effect of AIF deficiency on satellite cell clonogenicity, cells were plated at low density and the number of cells per clone was counted. The size of *Hq* microcolonies is altered compared to WT controls especially three days after plating. Indeed 95% of *Hq* myogenic colonies were mainly composed by one or two MyoD positive cells whereas about 70% WT myogenic colonies were already constituted by at least three cells (Fig. 7C). Four days after plating, the distribution of myogenic microcolonies constituted by more than three cells was increased in *Hq* primary cultures and the total number of *Hq* colonies was similar at day 3 and day 4, suggesting that *Hq* single cells observed at day 3 probably entered cell cycle between day 3 and day 4. These data propose that *Hq* satellite cells could enter later cell cycle in culture compared to WT satellite cells, suggesting that the activation step of *Hq* satellite cells might be longer than that of WT cells.

Cultured *Hq* mpc plated at high density were able to form proper contracting, sarcomeric MyHC positive myotubes (Fig. 7D). The diameter of newly formed myotubes was not significantly different in WT and *Hq* primary cultures (Fig. 7E). No significant difference in the number of nuclei per myotube was observed in these cultures (Fig. 7F), indicating that the impaired regeneration observed in *Hq* soleus and EDL muscle is not due to a differentiation defect of *Hq* myoblasts.

Combined, these data indicate that the impaired regenerative potential of *Hq* skeletal muscle most likely derives from a reduction in the satellite cell pool and an apparently abnormally long activation step of these cells.

## Discussion

### AIF regulates skeletal muscle physiology

The present set of data point towards an important role for AIF in several aspects of skeletal muscle physiology. Indeed, we confirmed that AIF is vital for the maintenance of skeletal muscle fiber size, since mice harboring the harlequin (*Hq*) mutation, leading to a>90% decrement in AIF expression, as mice with selective targeted deletion of *aif* in striated muscle, develop a severe form of atrophy [27]. Skeletal muscle atrophy is associated either with a decrease in the cytoplasmic content and /or with an impairment of myoblast cell fusion characterized by a loss of nuclei [42], [43]. Muscle hypertrophy is often correlated with an increase in myofiber nuclei [44], [45]. Many studies report that apoptosis is abundant in skeletal muscle during atrophy, suggesting that nuclei could be lost by apoptosis during atrophy. However, there is no evidence that these apoptotic nuclei are myonuclei (reviewed in [34]). Furthermore, a recent study using in vivo time lapse imaging technique revealed that myofibers do not lose myonuclei during muscle atrophy induced by a denervation or unloading [46]. Here, we report an example of skeletal muscle atrophy associated with a loss of myonuclei, as described in figure 1H. However, we were not able to observe apoptotic nuclei in atrophic *Hq* muscles nor in WT muscles, using TUNEL and anti-activated-caspase-3 staining (Fig. S1). Therefore, the loss of myonuclei observed in *Hq* muscles is probably not induced by apoptosis. Satellite cells are thought to serve as the source of myonuclei. When stimulated, satellite cells become activated and undergo cell division, after which they fuse into the existing muscle fiber [18]. It is likely that satellite cell proliferation is required for hypertrophy [47] and recovery from atrophy [48]. However, proliferation of satellite cell derived myoblasts is not affected in AIF deficient mice (Fig. 7) and therefore could not be involved in the atrophy observed in *Hq* muscles. One possible explanation for myonuclei loss in these muscles could be an impairment of myoblast fusion during developmental or adult myogenesis. This does not occur in *Hq* muscles since primary *Hq* myoblasts differentiate into myotubes with a number of nuclei similar to WT myotubes (Fig. 7), demonstrating that AIF deficiency does not affect skeletal muscle cell fusion nor differentiation. So, we think that the loss of myonuclei within myofibers, as well as the reduction in the satellite cell pool in *Hq* muscles, might be the consequence of a developmental problem in generating the correct amount of myoblasts, associated with an abnormal activation of some satellite cells during adulthood (Fig. 6). It could be very interesting to study AIF deficiency and therefore increased oxidative stress during embryonic and postnatal development.

Our data also point to an additional role for AIF in fiber type determination of skeletal muscle, since skeletal muscle from *aif* hypomorphic harlequin mutant mice preferentially expressed the slowest forms of MyHC in soleus and EDL hindlimb muscle, and this pattern of preferential “slow” fiber type expression was maintained even following cardiotoxin-induced regeneration. Muscle fibers are generally characterized as being oxidative/slow (expressing primarily type I myosin heavy chain [MyHC]), intermediate, or glycolytic/fast (expressing type IIa/x/b MyHC), and fiber type determination is dictated by slow/fast motoneuron activity [49]. Given the neurodegenerative defects of *Hq* mutant mice, we scrutinized the integrity of neurotransmission in *Hq* mice by analyzing the neuromuscular junction, which we confirmed to be intact (Fig. S2). In addition, Klein and coworkers demonstrated that AIF deficiency provokes progressive degeneration of specific cerebellar and retinal neurons in adulthood [24] and during development [50], but observed no neuron loss in the cerebral motor cortex, even though AIF is expressed in this cerebral region. We cannot exclude, however, that motor neurons or their activity are not affected in *Hq* mutant mice, which could contribute to the skeletal muscle atrophy and fiber type switch, given that the diameter of newly formed myotubes was not significantly different in WT and *Hq* primary cultures (Fig. 7D, 7E).

Alternatively, the enhanced oxidative stress in AIF deficient myofibers could also contribute to the fiber-type switch in *aif* hypomorphic mice. The fiber-type program of adult skeletal muscle is highly plastic and dynamically regulated by motor nerve activity, which is associated with altered intracellular calcium handling, and contingent upon the activation of specific intracellular signaling pathways. One such pathway utilizing the calcium-activated posphatase calcineurin, selectively activates expression of MyHC I [51] and stimulates transcription from slow fiber-specific gene promoters partially through the activation of MEF2 transcription factors [39], [40]. Oxidative stress modulates calcium transients [52], which may enhance calcineurin and MEF2 activity indirectly (Fig. 3G), and provoke the activation of slow fiber-specific genes. Additionally, oxidative stress modulates the phosphorylation status and calcineurin-inhibitory activities of DSCR1 (Adapt78/MCIP1) protein [53], a calcineurin accessory protein. Conclusively, the enhanced oxidative stress in *aif* hypomorphic muscle fibers might contribute to a switch in MyHC expression by indirectly modulating calcineurin and MEF2 activity in a cell autonomous manner.

### AIF regulates the skeletal muscle precursor pool and self-renewal

Loss of muscle mass, or sarcopenia, a common observation in aging muscles, is directly related to a significant reduction of the regenerative potential of muscle [54]. In humans, skeletal muscle stem cells display a steady decline in replicative capacity and muscle-specific gene expression with increasing donor age [55], [56]. Moreover, the absolute number of satellite cells also decreases with age [13], whereas their ability to proliferate and differentiate seems to be retained [56]. Oxidative stress has been suggested to play a role in satellite cell self-renewal and function [9]. We demonstrate here that oxidative stress associated with AIF deficiency suffices to provoke a reduction in the number of satellite cells and probably to lengthen their activation step, which we could directly link to the observed limitation in muscular regenerative capacity. The in vivo increase in activated satellite cells in *Hq* muscles (Fig. 6H) could reflect the longer activation step observed in vitro (Fig. 7). It seems that these in vivo activated satellite cells cannot go further in their activation step since these cells were not more active in the in vitro clonogenicity test. Based upon M-cadherin staining, the satellite cell pool was reduced to the same extent in both *Hq* soleus and EDL muscles (Fig. 6F). Factors that govern satellite cell frequency in muscles are not known. But, several observations show that type I fibers are associated to more satellite cells than type II fibers [57], and this correlation seems to depend on their capillarization [58], which could suggest a role of oxidative stress. M-cadherin positive cells were indeed more abundant in the “slow” muscle, soleus, than in the “fast” EDL muscle in *Hq* mice as in the WT ones (Fig. 6F). So, oxidative stress induced by AIF deficiency affects EDL satellite cells as it does with soleus ones and its implication in *Hq* satellite cell deficiency seems to be fiber type independent. M-cadherin is a marker of a subset of satellite cells, whereas Pax7 is expressed by most of satellite cells. In our experiments, Pax7 positive cells were less abundant than M-cadherin positive cells in one particular muscle, the soleus. This observation suggests two distinct satellite cell populations in this muscle: satellite cells that express high level of Pax7, and cells that appear as “Pax7 negative” or express Pax7 at such level of expression that we were not able to detect them by immunohistochemistry, as already described in human [59]. In *Hq* soleus muscle, the average number of Pax7 positive cells is similar to the number of M-cadherin positive cells showing that AIF deficiency did not alter this population of satellite cells, most likely co-expressing Pax7 and M-cadherin. The “Pax7 negative”, M-cadherin positive cells were then specifically affected in *Hq* soleus muscles. These observations strongly suggest the existence of a heterogenous satellite cell population, differentially sensitive to oxidative stress.

Given that antioxidant pretreatment fully rescued the satellite cell pool of *aif* hypomorphic myofibers, suggests that AIF plays a vital role as an antioxidant protecting stem cells against oxidative stress. A survey of various stem and progenitor cells indicates that these cells might have developed unique antioxidant defense mechanisms to cope with accumulative ROS load, to avoid oxidative stress-induced damage [60]. The data in the present manuscript suggest that AIF might be involved in this antioxidant defense, at least in satellite cells. Several syndromes like Ataxia telangiectasia mutated (ATM) are characterized by increased oxidative stress [14], and interestingly, the self-renewal of haematopoietic stem cells derived from ATM-deficient mice was impaired, ROS levels were increased in ATM-deficient haematopoietic cells, and bone-marrow failure was rescued by simple antioxidant treatment [14]. It will be of interest to analyze whether AIF participates in antioxidant protection in other stem cells than muscle progenitor cells, and in other mouse models of increased oxidative stress.

Among the stimuli that have been identified to evoke cellular and organism senescence, oxidative stress plays an important role [61]. The variety of phenotypes observed in *Hq* mice resembles those in patients with premature-aging syndromes: increased oxidative stress, accelerated progression to heart failure in response to stress, decreased skeletal muscle mass (Fig. 1I), skeletal muscle atrophy (Fig. 1E), a “fiber type switch from fast to slow” (Figs. 3, 5) impaired skeletal muscle regeneration (Fig. 4), with a poor skeletal stem cell pool (Fig. 6), neurodegenerative changes, lordokyphosis, age-related skin changes and alopecia (hair loss), growth retardation, and short lifespan. As such, the variety of syndromes in *Hq* mice suggest that this mutant could be a relevant model to study how oxidative stress induces premature-aging phenotypes, and the specific role of mitochondrial AIF in these processes.

## Supporting Information

Figure S1 **AIF deficiency does not affect adult muscle cell viability.** (A) LC3 staining revealed a granular appearance in one myofiber observed on an EDL section from a 2 month old *Hq* mouse. Nuclei were stained with Bis-benzimide. Bar =  µm. (B) Representative sections of WT and *Hq* EDL muscles from 2 month old mice, stained with anti-LC3 antibody and Bis-benzimide to reveal autophagic myofibers and total nuclei, respectively indicate an absence of autophagic myofibers in both WT and *Hq* EDL muscles. Bar = 50 µm. (C) Antibodies recognizing the activated form of caspase-3 reveals one apoptotic cell in one EDL *Hq* muscle. Nuclei were stained with Bis-benzimide. Bar = 25 µm. (D) Representative sections of WT and *Hq* EDL muscles from 2 month old mice, stained with anti- activated caspase 3 antibody and Bis-benzimide to reveal apoptotic myonuclei and total nuclei, respectively indicate that AIF deficiency is not associated to an increase of apoptotic cells. Bar = 50 µm. (E) Calcium deposition revealed by the Alizarin method occurs in necrotic fibers of WT soleus muscles 3 days following a cardiotoxin injection. No traces of calcium are seen in non-regenerating myofibers of 2 month old WT and *Hq* mice, indicating the absence of necrotic fibers in *Hq* muscles. Bar = 100 µm.
(TIF)

Figure S2 **Normal neuromuscular junctions in** ***Hq*** **skeletal muscles.** (A–D) Representative pictures of neuromuscular junctions of soleus (A, B) and EDL (C, D) muscles from WT (A, C) and *Hq* (B, D) mice were immunostained to visualize acetylcholine (Ach) receptor distribution. Bar = 7 µm.
(TIF)
