# Supplementary figures and images for: Apoptosis-Inducing Factor Regulates Skeletal Muscle Progenitor Cell Number and Muscle Phenotype
Source: PLoS One. 2011 Nov 4;6(11):e27283. doi: 10.1371/journal.pone.0027283 (PMC3208607; doi:10.1371/journal.pone.0027283)

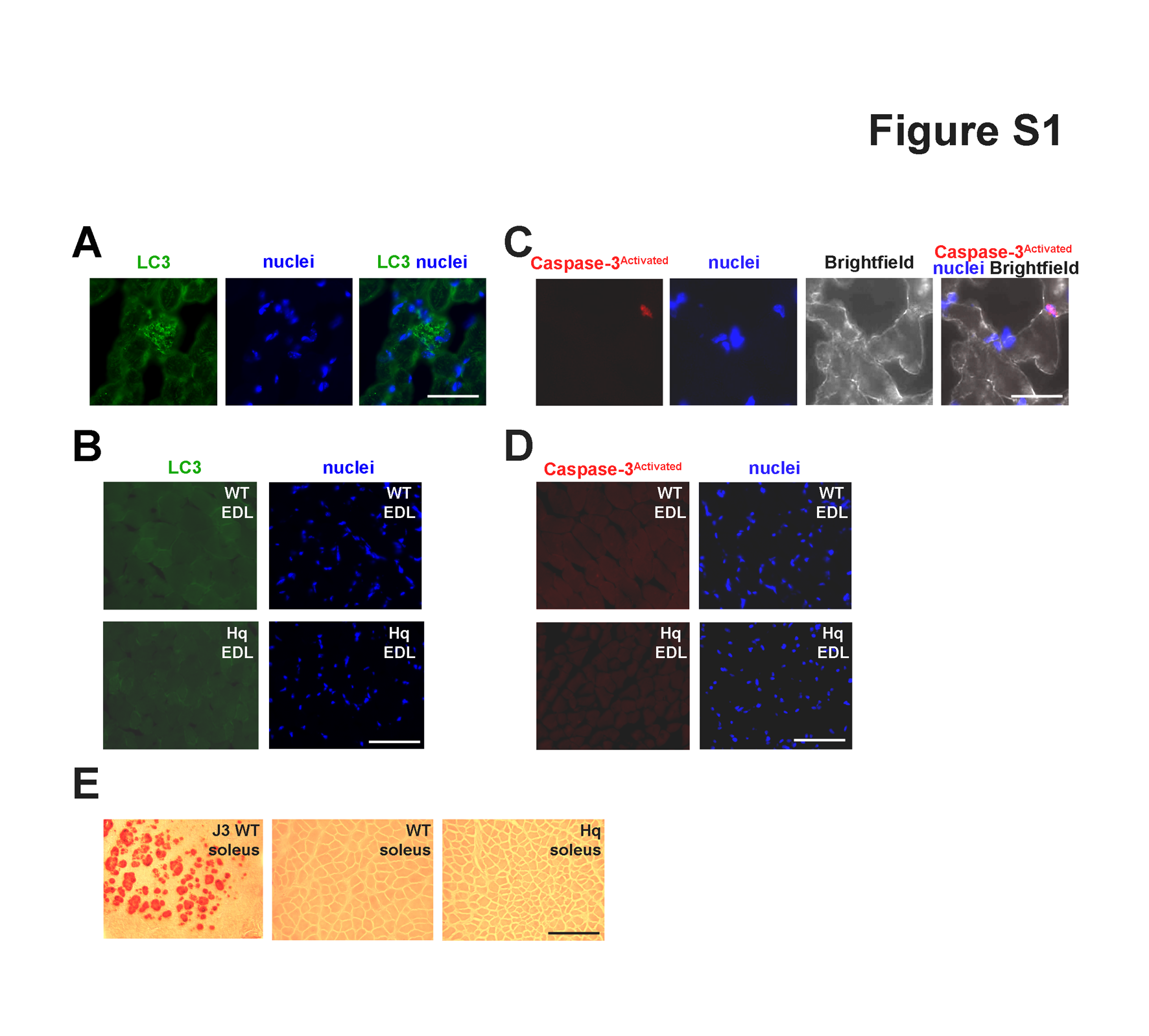

Supplement: Figure S1 — AIF deficiency does not affect adult muscle cell viability. (A) LC3 staining revealed a granular appearance in one myofiber observed on an EDL section from a 2 month old Hq mouse. Nuclei were stained with Bis-benzimide. Bar = µm. (B) Representative sections of WT and Hq EDL muscles from 2 month old mice, stained with anti-LC3 antibody and Bis-benzimide to reveal autophagic myofibers and total nuclei, respectively indicate an absence of autophagic myofibers in both WT and Hq EDL muscles. Bar = 50 µm. (C) Antibodies recognizing the activated form of caspase-3 reveals one apoptotic cell in one EDL Hq muscle. Nuclei were stained with Bis-benzimide. Bar = 25 µm. (D) Representative sections of WT and Hq EDL muscles from 2 month old mice, stained with anti- activated caspase 3 antibody and Bis-benzimide to reveal apoptotic myonuclei and total nuclei, respectively indicate that AIF deficiency is not associated to an increase of apoptotic cells. Bar = 50 µm. (E) Calcium deposition revealed by the Alizarin method occurs in necrotic fibers of WT soleus muscles 3 days following a cardiotoxin injection. No traces of calcium are seen in non-regenerating myofibers of 2 month old WT and Hq mice, indicating the absence of necrotic fibers in Hq muscles. Bar = 100 µm. (TIF) [file pone.0027283.s001.tif]

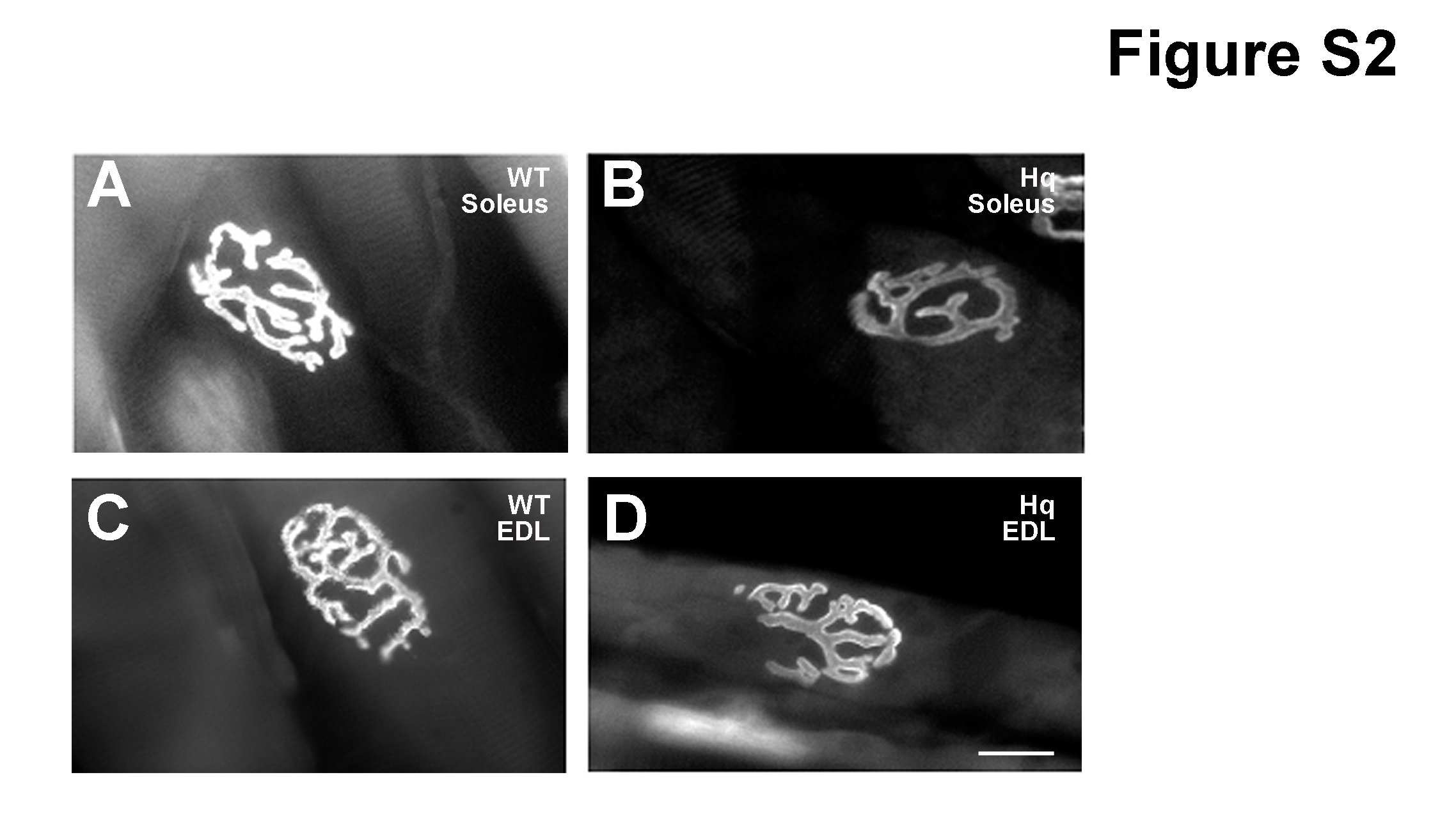

Supplement: Figure S2 — Normal neuromuscular junctions in Hq skeletal muscles. (A–D) Representative pictures of neuromuscular junctions of soleus (A, B) and EDL (C, D) muscles from WT (A, C) and Hq (B, D) mice were immunostained to visualize acetylcholine (Ach) receptor distribution. Bar = 7 µm. (TIF) [file pone.0027283.s002.tif]
